# Supplementary figures and images for: Liver microRNA transcriptome reveals miR-182 as link between type 2 diabetes and fatty liver disease in obesity
Source: eLife. 2024 Jul 22;12:RP92075. doi: 10.7554/eLife.92075 (PMC11262792; doi:10.7554/eLife.92075)

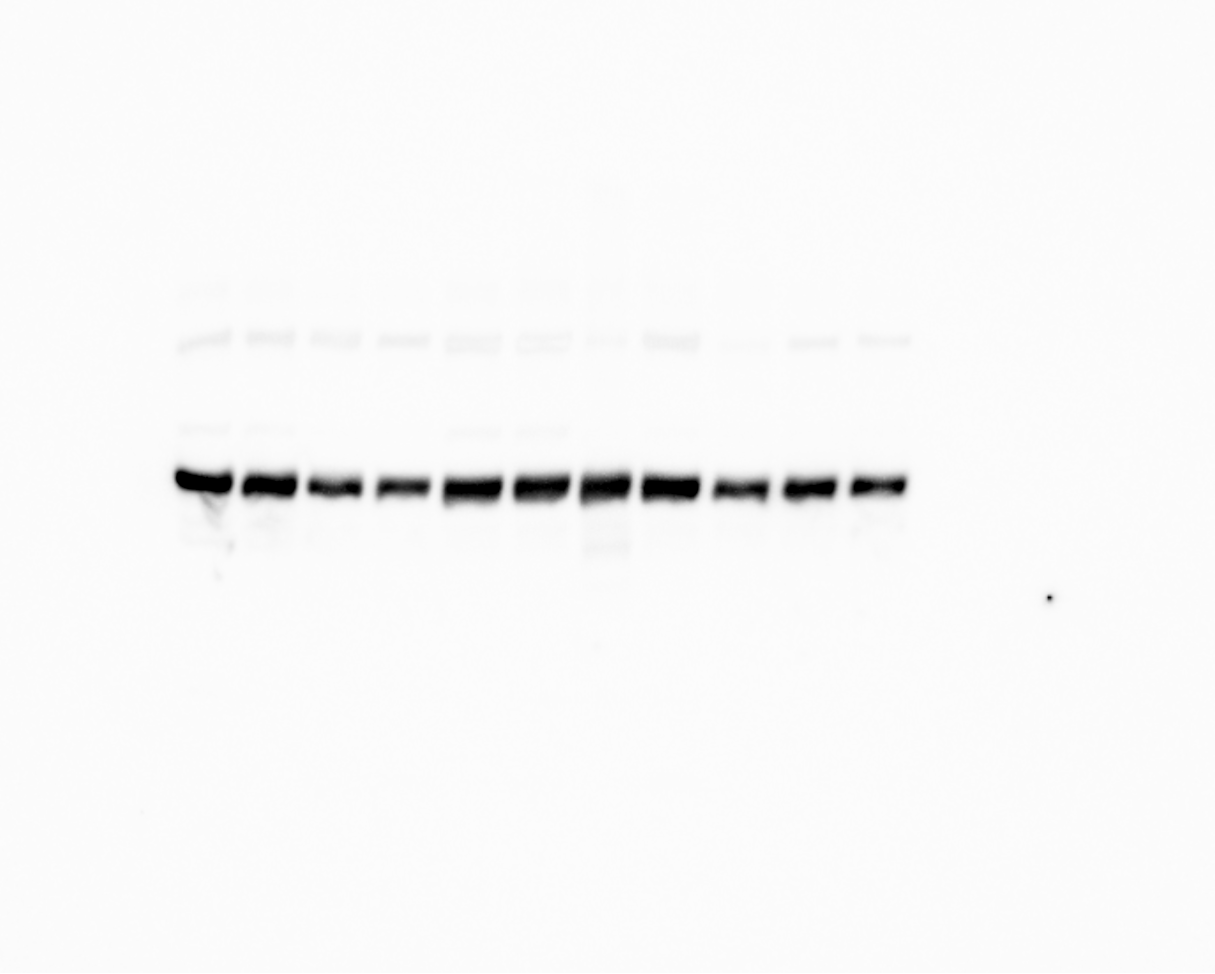

Supplement: Figure 2—source data 1. [file elife-92075-fig2-data1.zip › Figure 2E Replicate 1 and 2/HSP90 AG Kirchner 2020-10-23 13h19m23s(Chemiluminescence).tif]

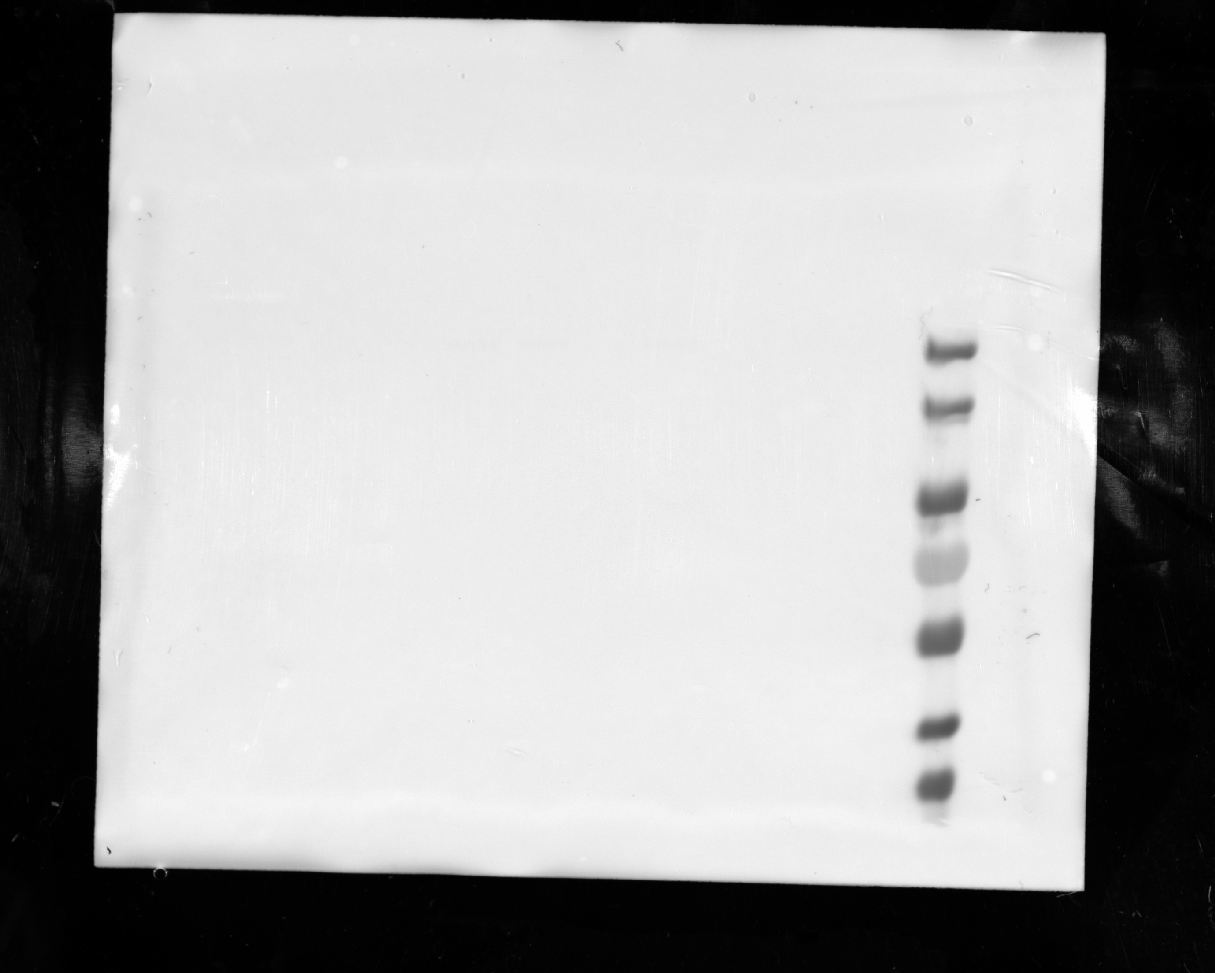

Supplement: Figure 2—source data 1. [file elife-92075-fig2-data1.zip › Figure 2E Replicate 1 and 2/HSP90 Marker AG Kirchner 2020-10-23 13h18m28s(Colorimetric).tif]

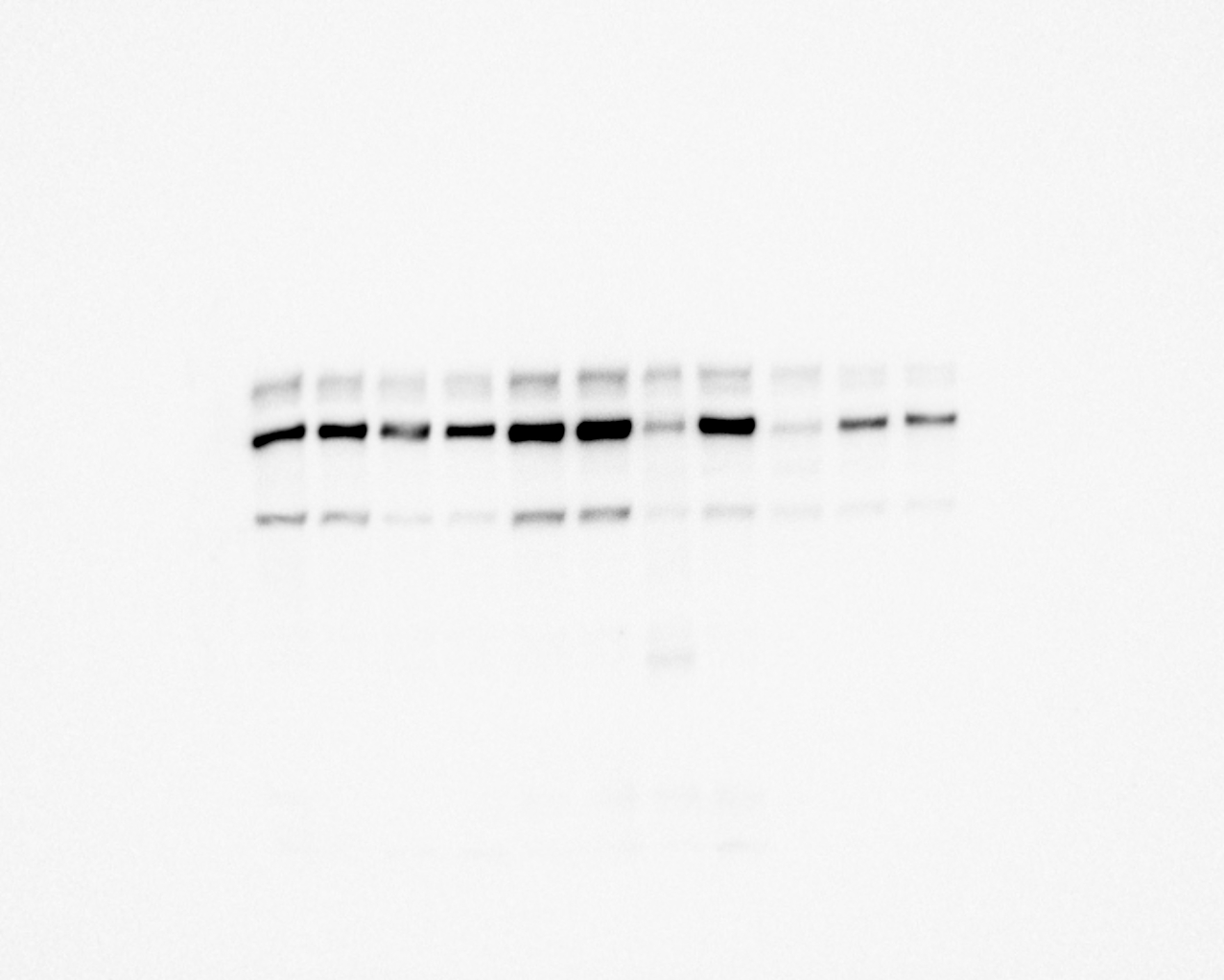

Supplement: Figure 2—source data 1. [file elife-92075-fig2-data1.zip › Figure 2E Replicate 1 and 2/LRP6 AG Kirchner 2020-10-22 13h18m05s(Chemiluminescence).jpg]

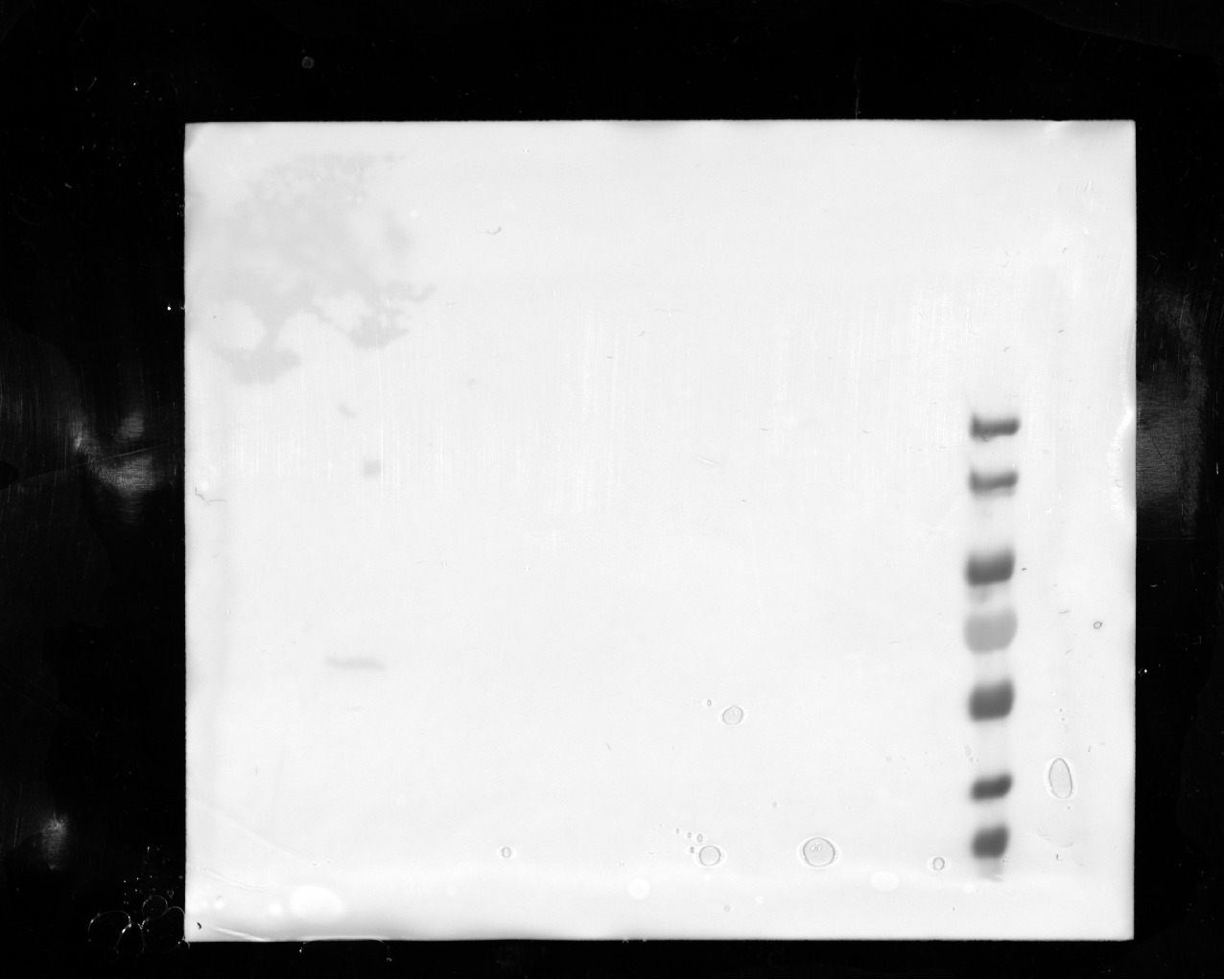

Supplement: Figure 2—source data 1. [file elife-92075-fig2-data1.zip › Figure 2E Replicate 1 and 2/LRP6 Marker AG Kirchner 2020-10-22 13h17m23s(Colorimetric).tif]

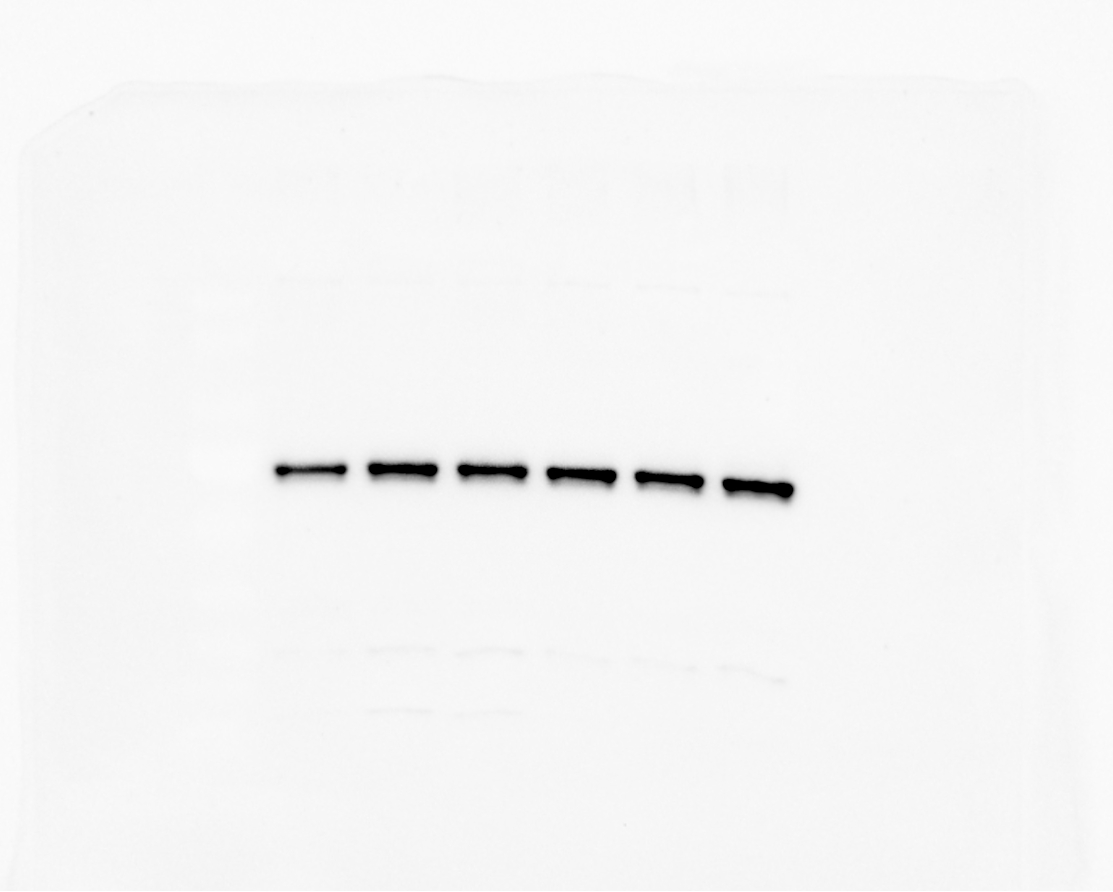

Supplement: Figure 2—source data 3. [file elife-92075-fig2-data3.zip › Figure 2H Replicate 1 Akt/AG Kirchner 2020-12-04~WB-Versuch2~AktAk(Chemiluminescence).jpg]

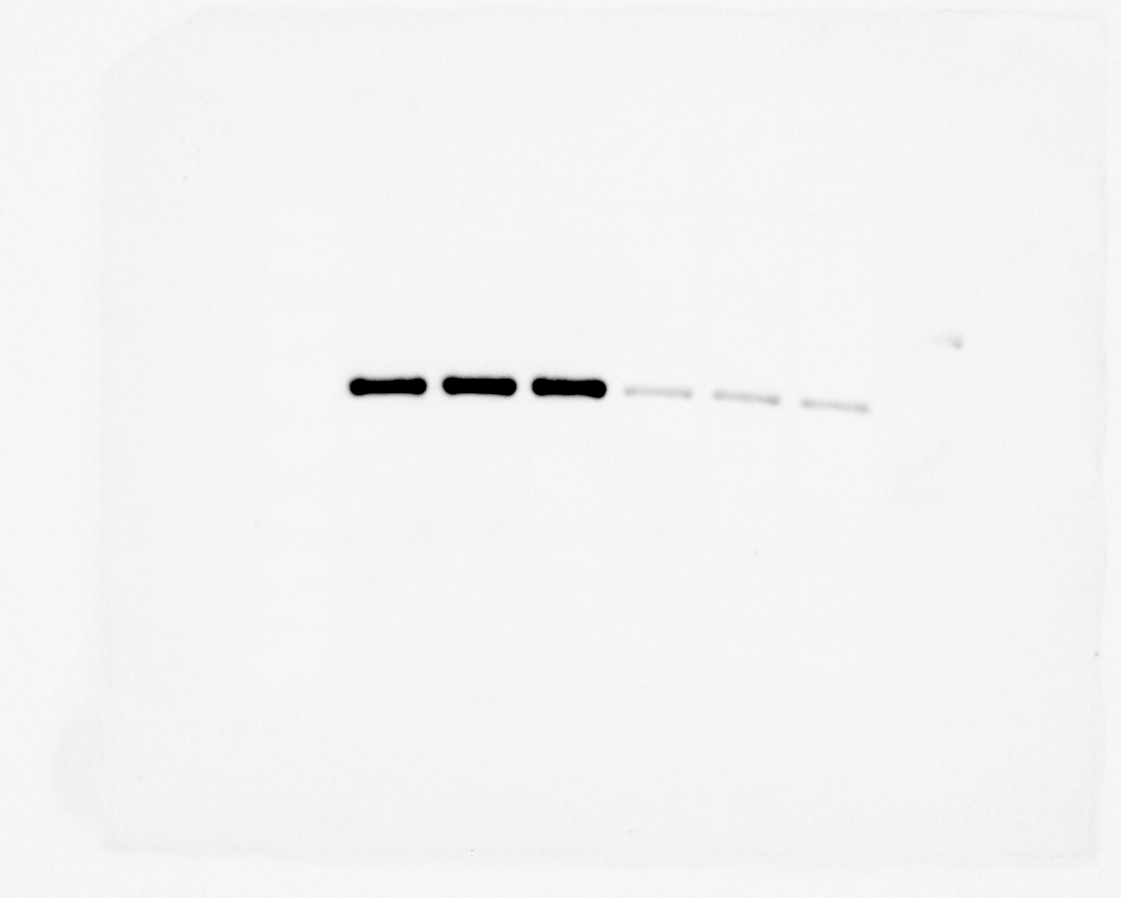

Supplement: Figure 2—source data 3. [file elife-92075-fig2-data3.zip › Figure 2H Replicate 1 pAkt/AG Kirchner 2020-12-03-WB-Versuch2-PhosphoAkt(Chemiluminescence).jpg]

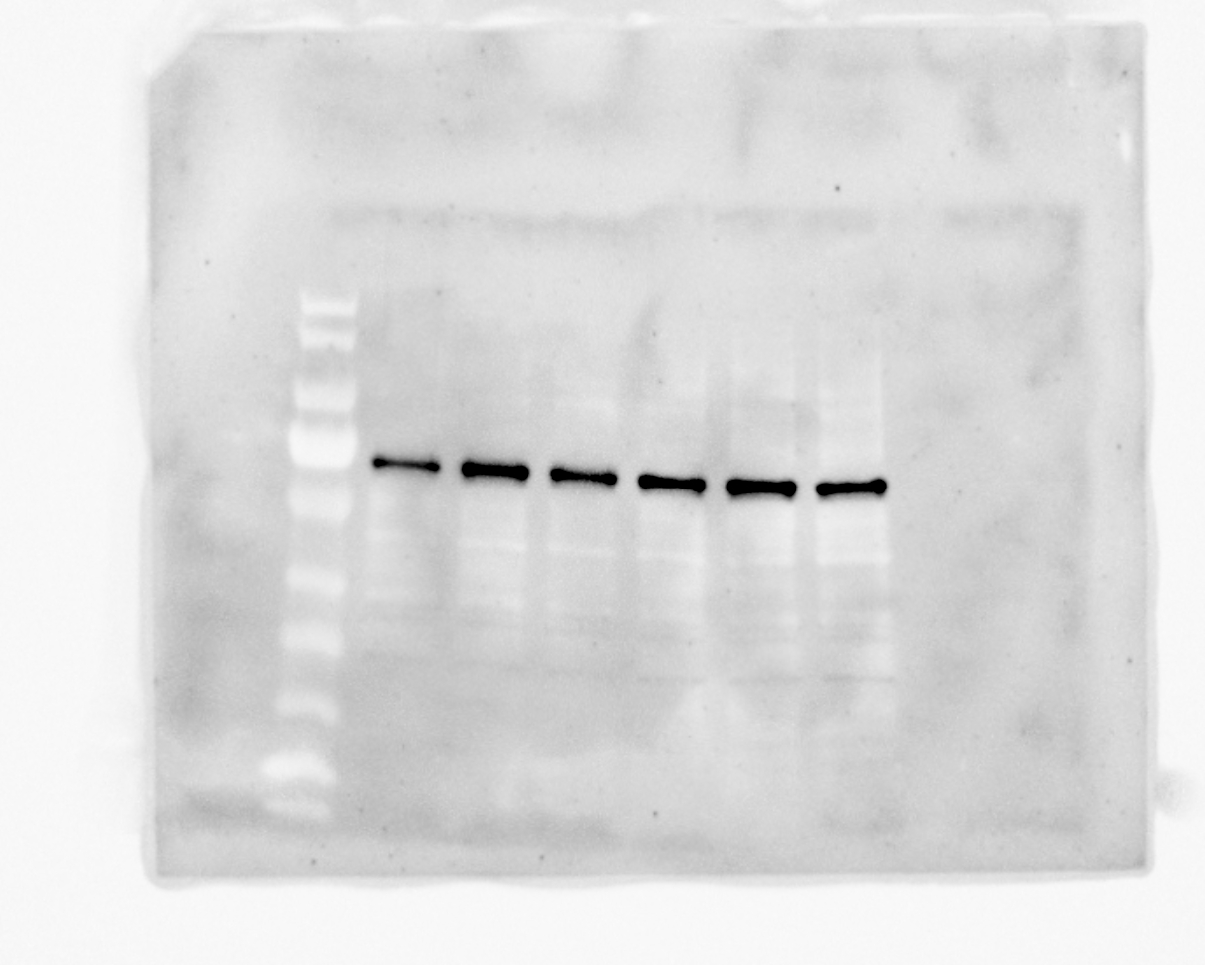

Supplement: Figure 2—source data 3. [file elife-92075-fig2-data3.zip › Figure 2H Replicate 2 Akt/AG Kirchner 2020-11-27-AktAK-WB4(Chemiluminescence).jpg]

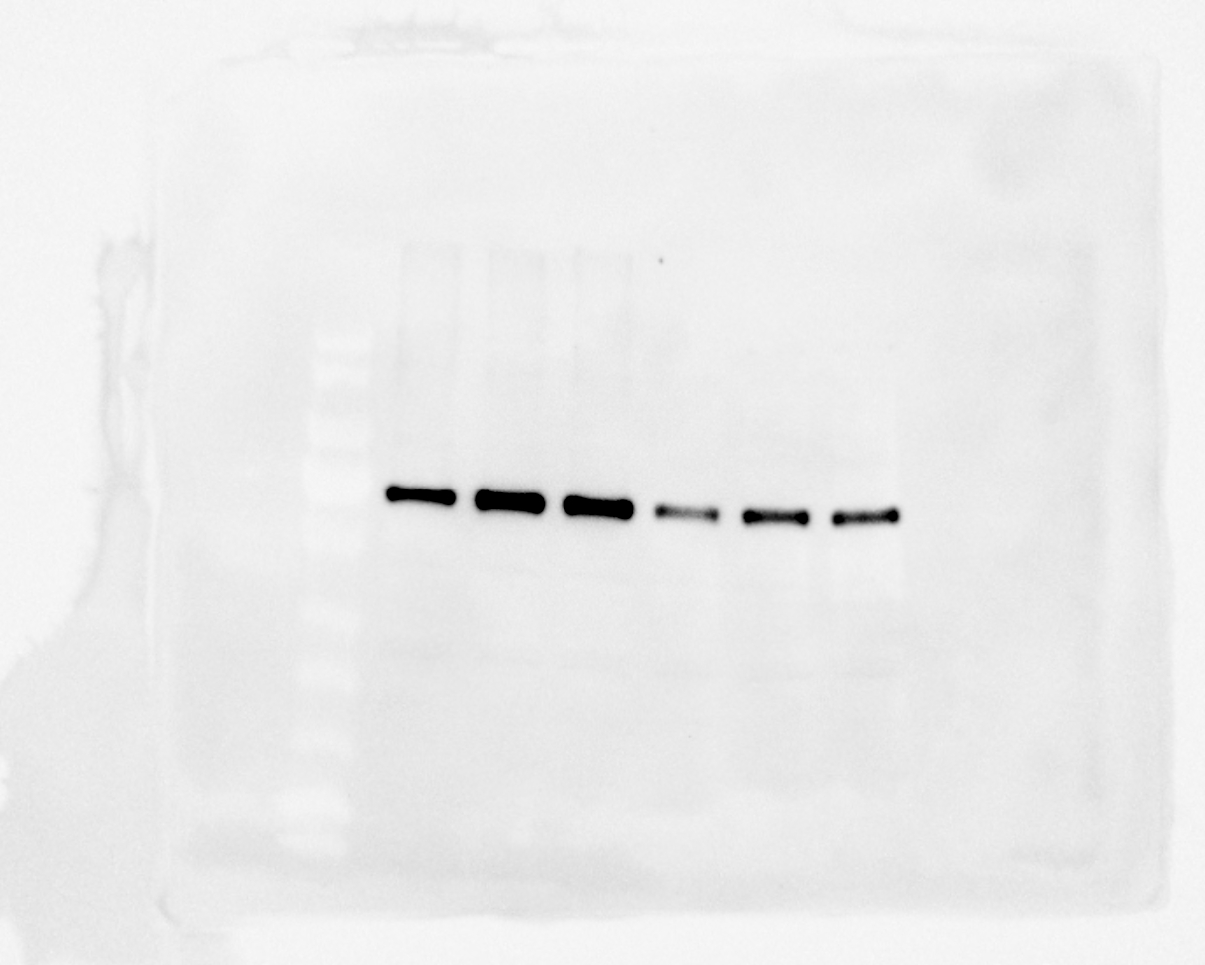

Supplement: Figure 2—source data 3. [file elife-92075-fig2-data3.zip › Figure 2H Replicate 2 pAkt/AG Kirchner 2020-11-26-PhoshpoAkt-WB4(Chemiluminescence).jpg]

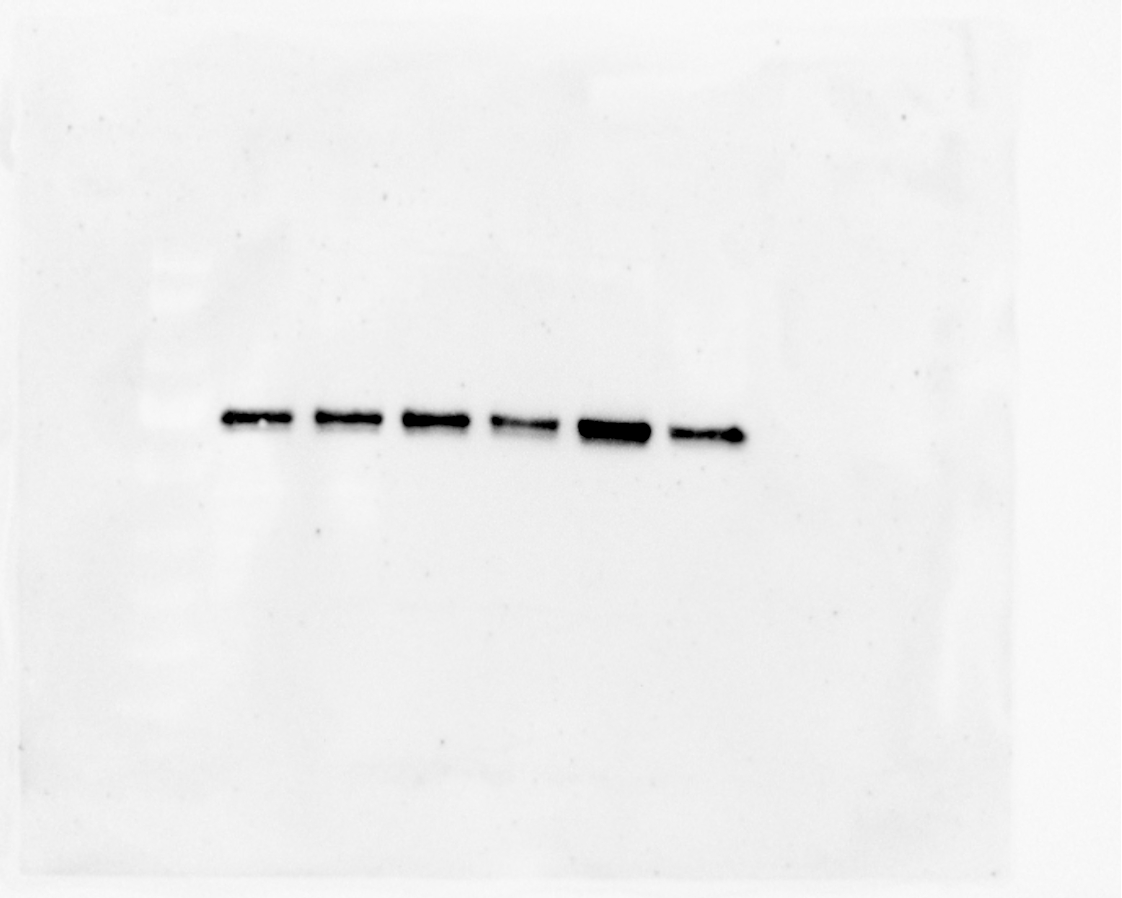

Supplement: Figure 2—source data 3. [file elife-92075-fig2-data3.zip › Figure 2H Replicate 3 Akt/AG Kirchner 2020-10-15-AktAK-WB1(Chemiluminescence).jpg]

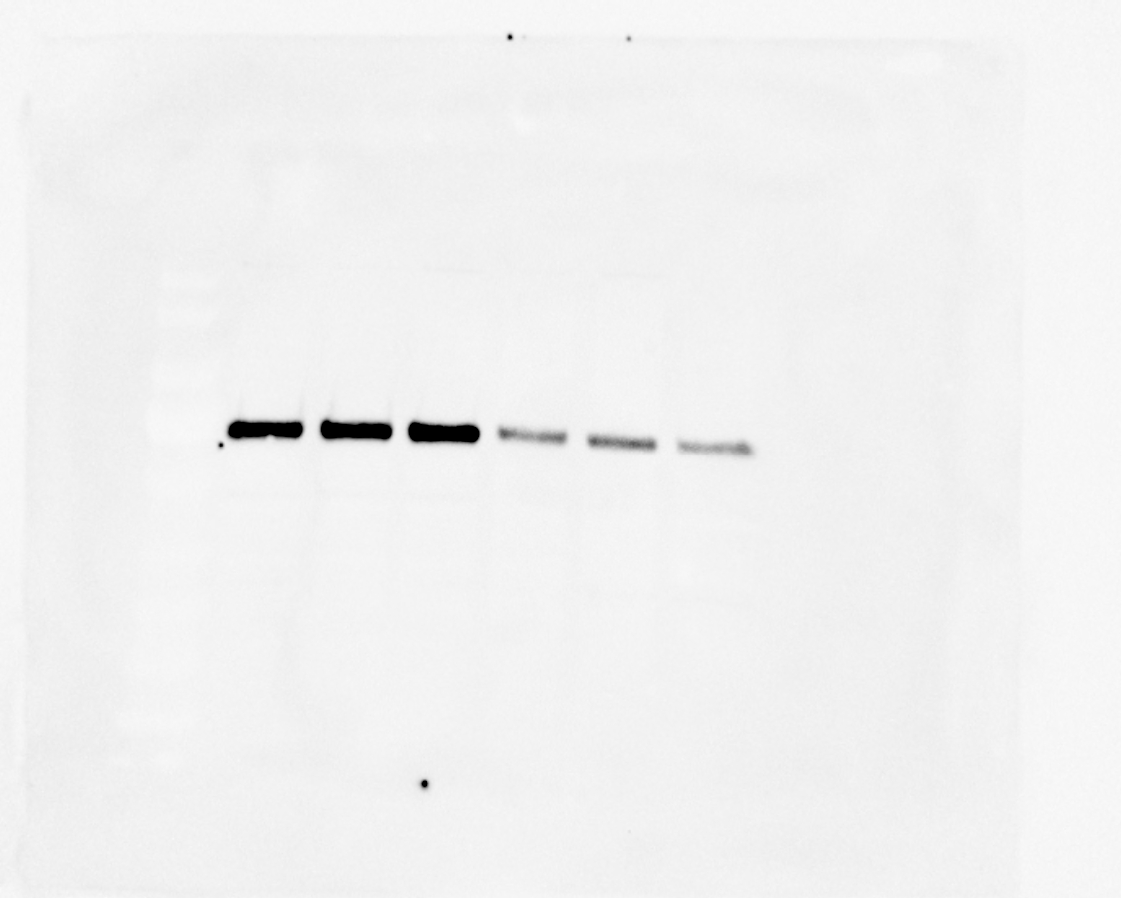

Supplement: Figure 2—source data 3. [file elife-92075-fig2-data3.zip › Figure 2H Replicate 3 pAkt/AG Kirchner 2020-10-14-PhosphoAkt-WB1(Chemiluminescence).jpg]

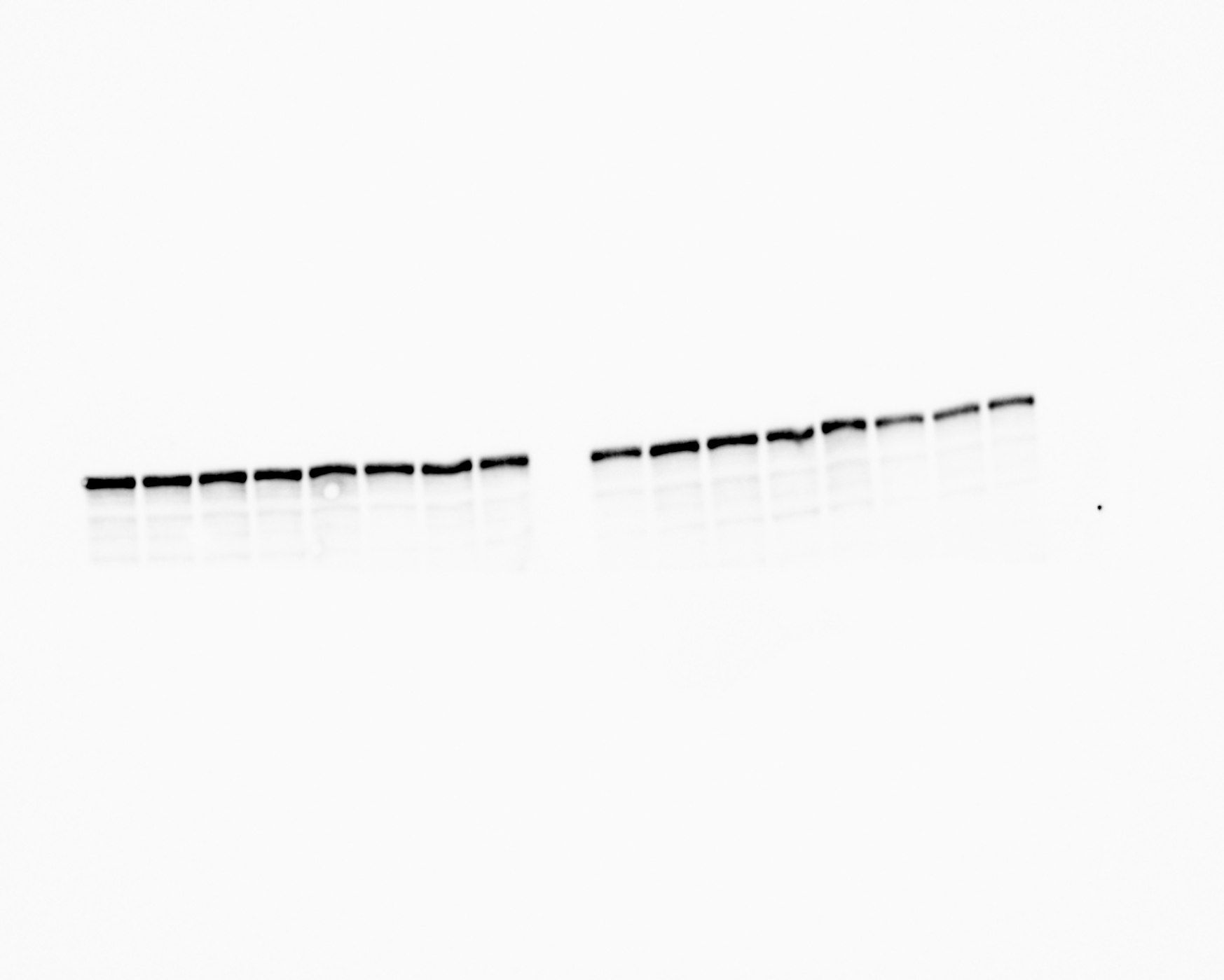

Supplement: Figure 4—source data 1. [file elife-92075-fig4-data1.zip › Figure 4J Western Blots Lrp6 und Hsp90 mouse trial/Hsp90_chemiluminescence.jpg]

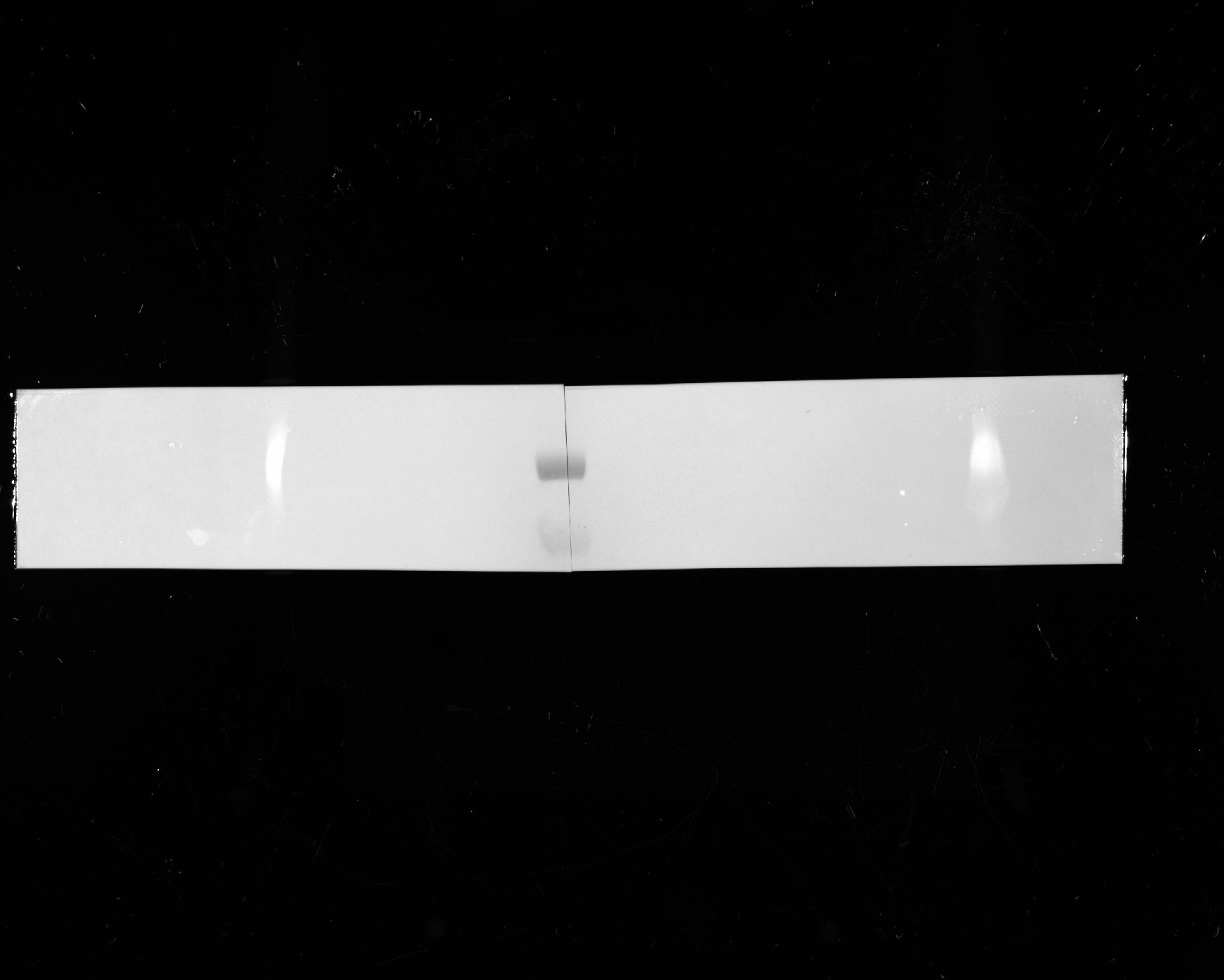

Supplement: Figure 4—source data 1. [file elife-92075-fig4-data1.zip › Figure 4J Western Blots Lrp6 und Hsp90 mouse trial/Hsp90_colorimetric.jpg]

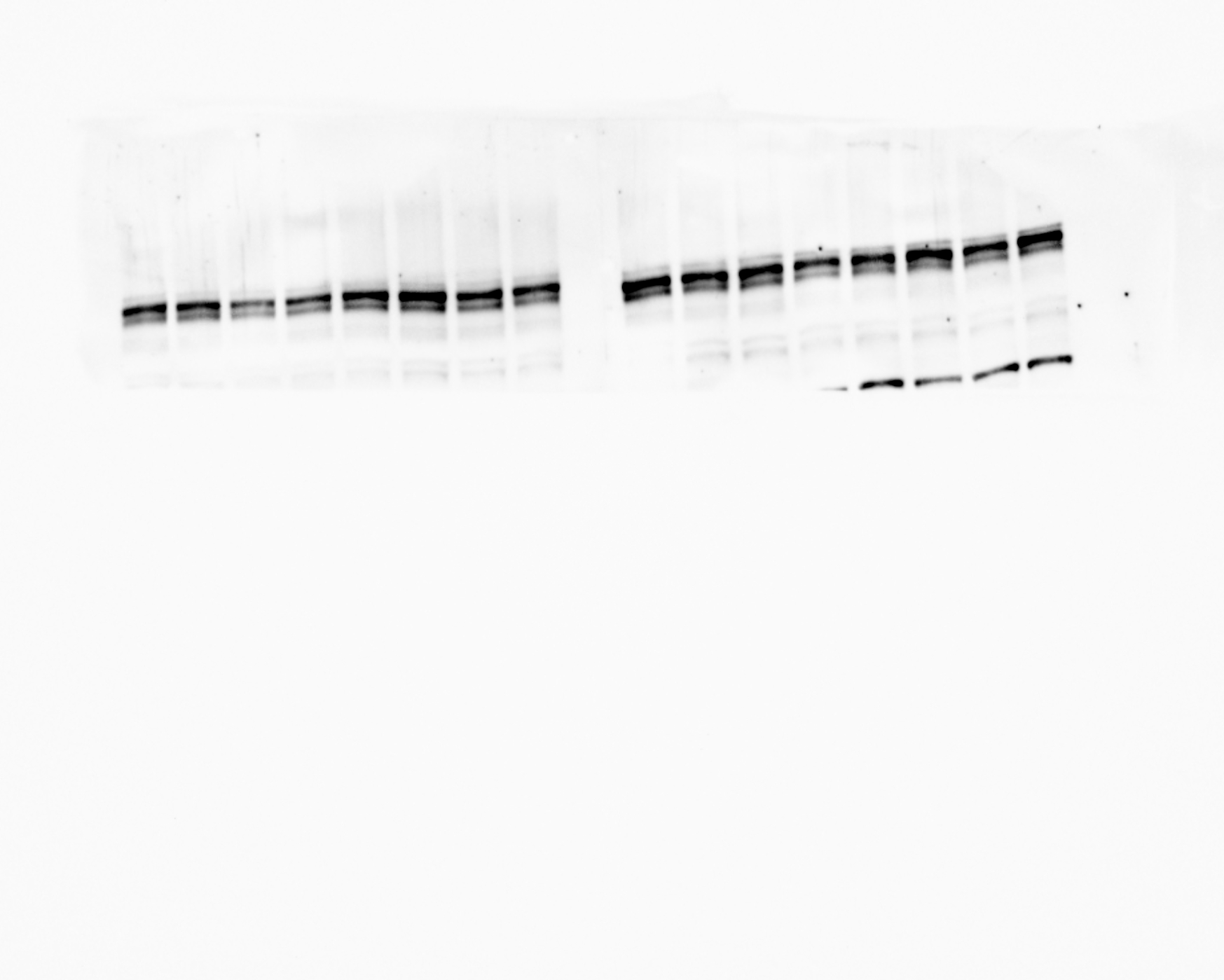

Supplement: Figure 4—source data 1. [file elife-92075-fig4-data1.zip › Figure 4J Western Blots Lrp6 und Hsp90 mouse trial/Lrp6_chemiluminescence.jpg]

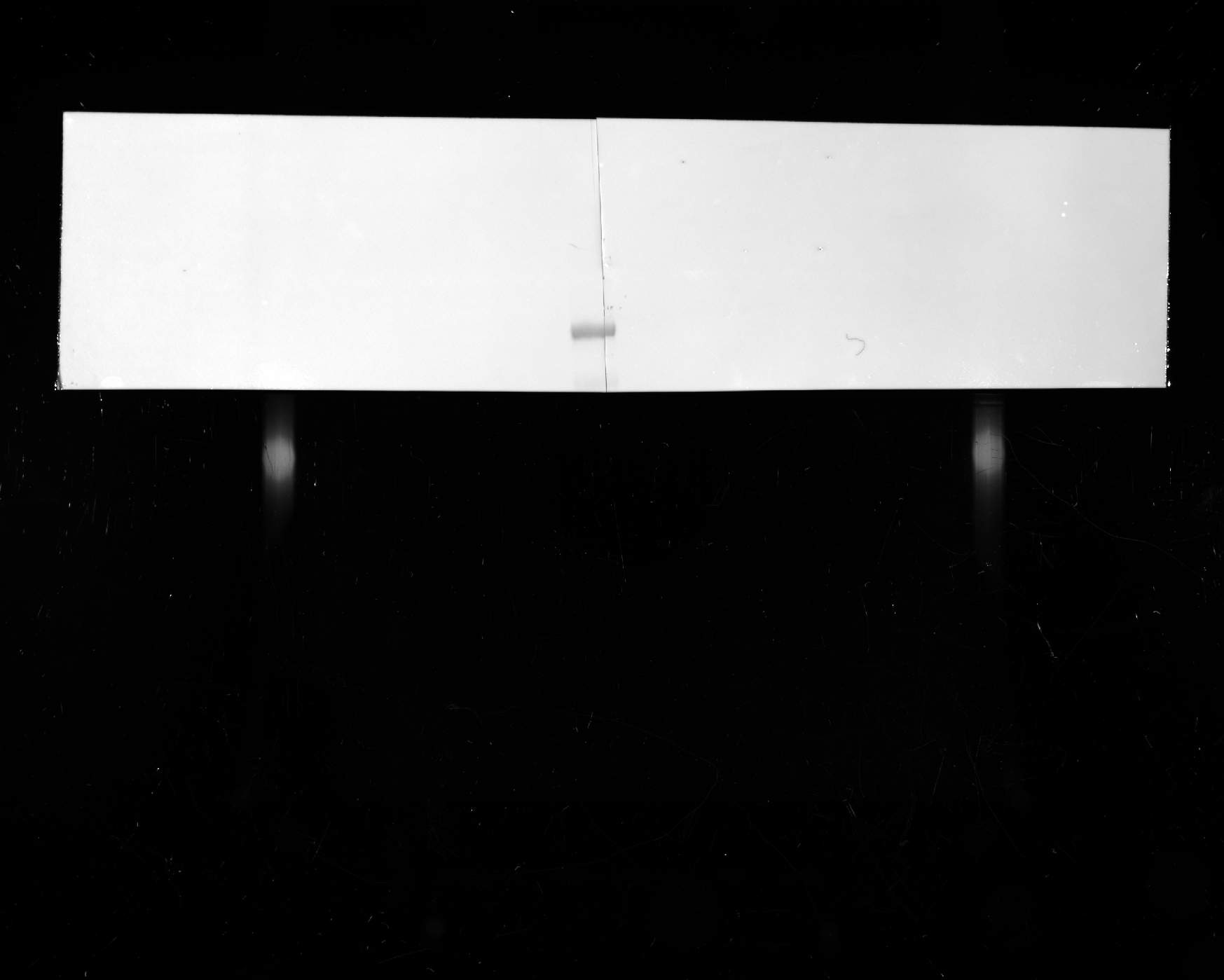

Supplement: Figure 4—source data 1. [file elife-92075-fig4-data1.zip › Figure 4J Western Blots Lrp6 und Hsp90 mouse trial/Lrp6_colorimetric.jpg]

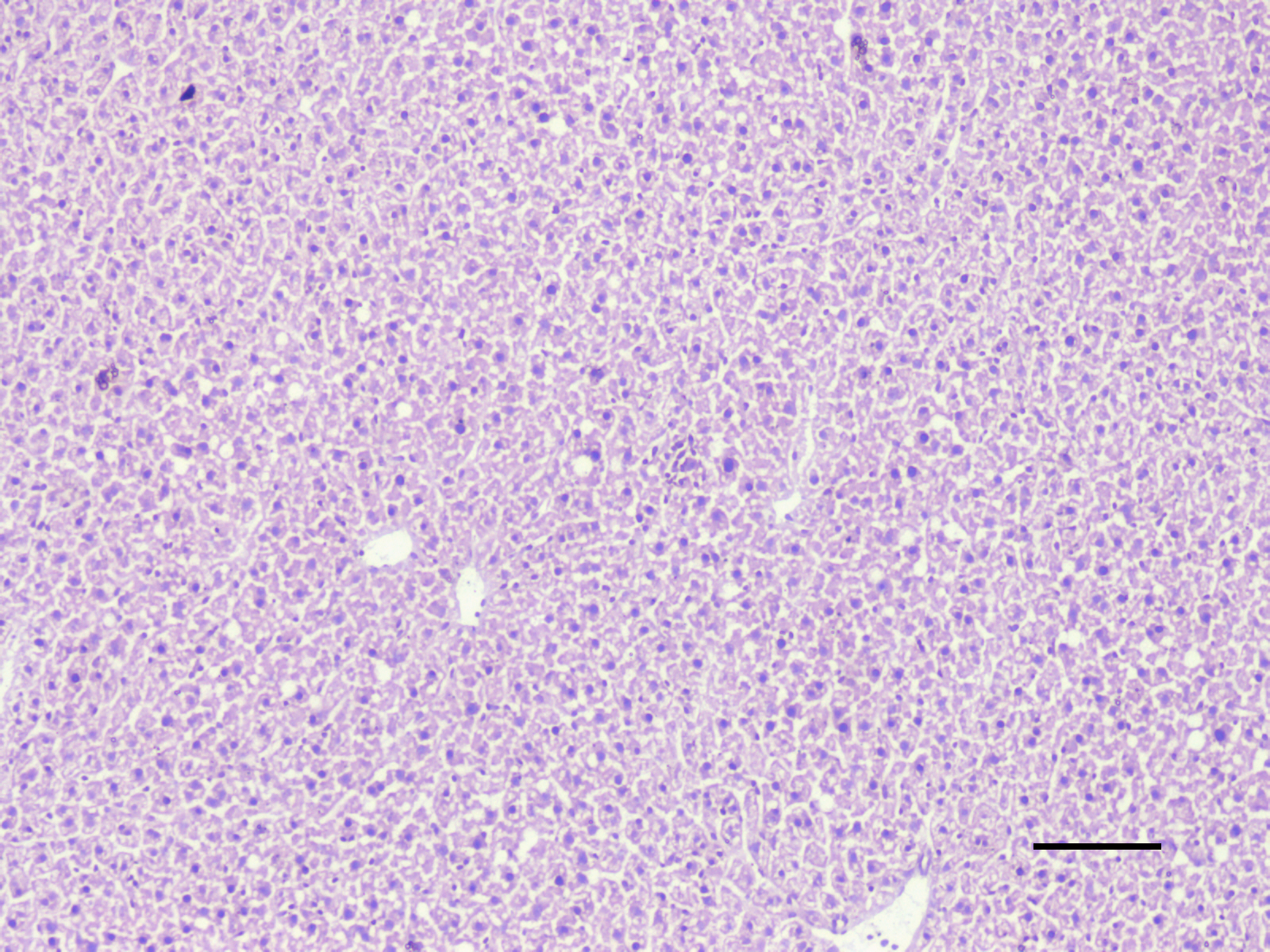

Supplement: Figure 4—figure supplement 1—source data 1. [file elife-92075-fig4-figsupp1-data1.zip › miR23_miR182_10x_1_scale.jpg]

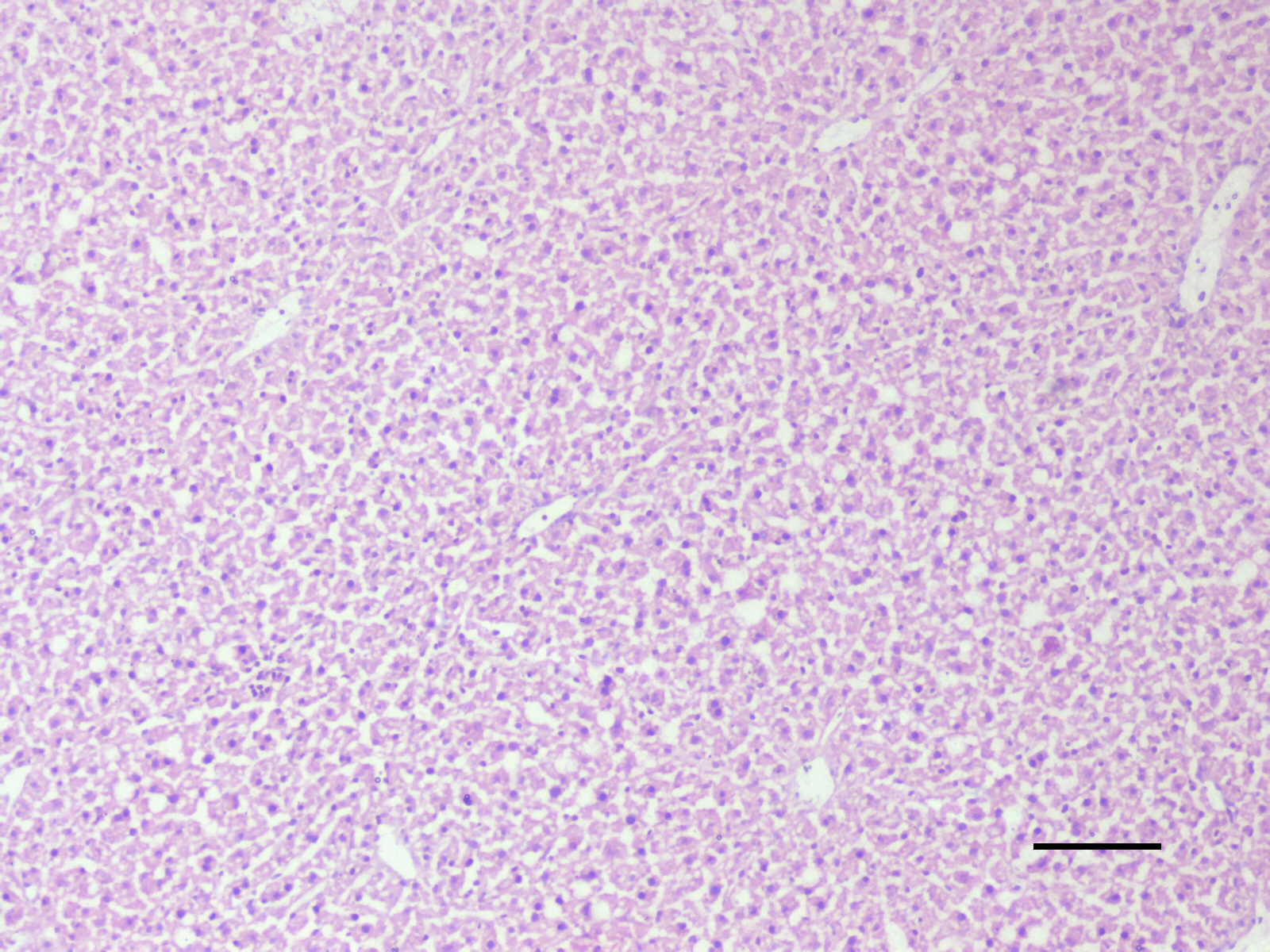

Supplement: Figure 4—figure supplement 1—source data 1. [file elife-92075-fig4-figsupp1-data1.zip › miR30_miR182_10x_3_scale.jpg]

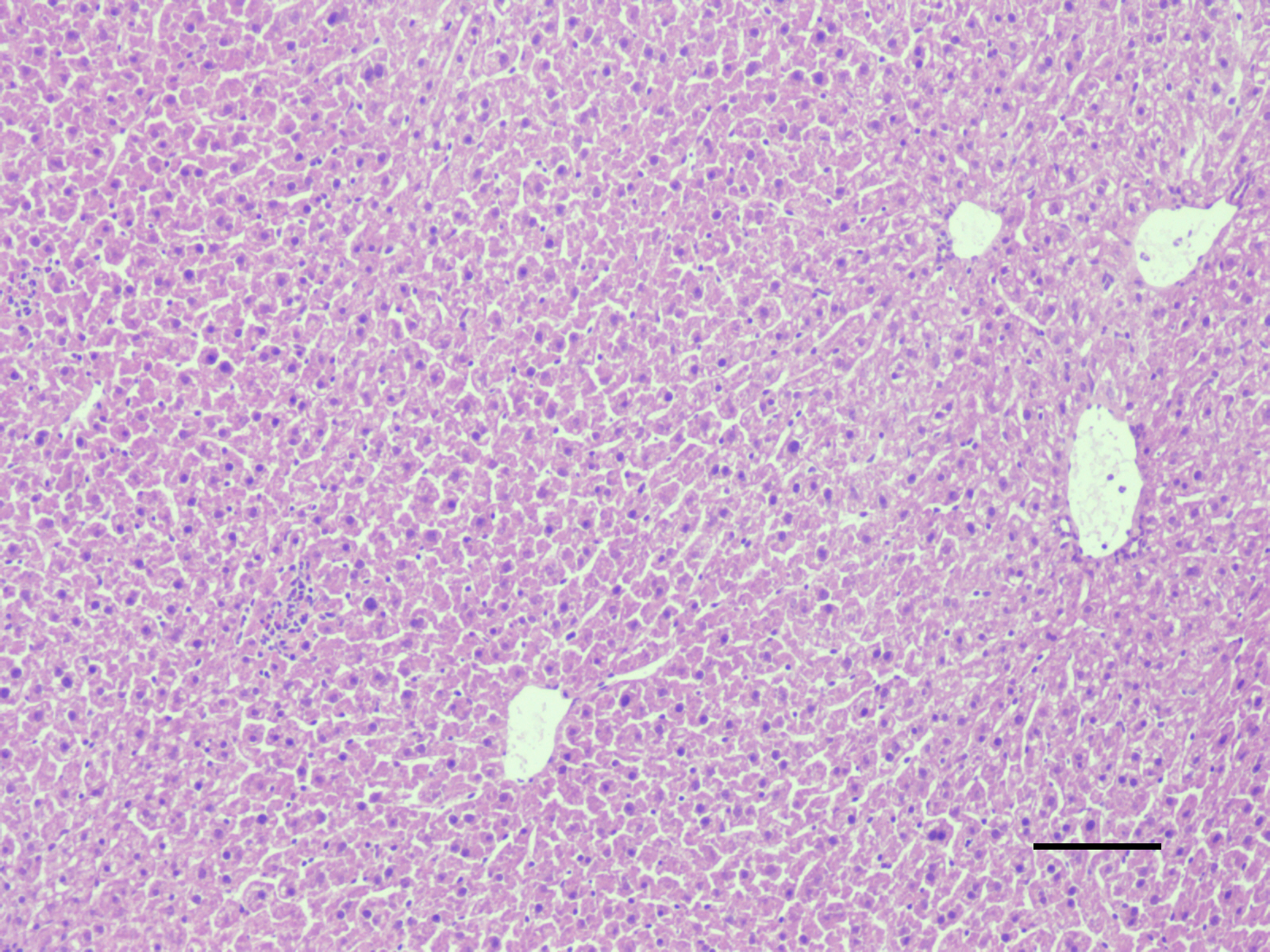

Supplement: Figure 4—figure supplement 1—source data 1. [file elife-92075-fig4-figsupp1-data1.zip › miR32_10x_ID_43_3_scale.jpg]

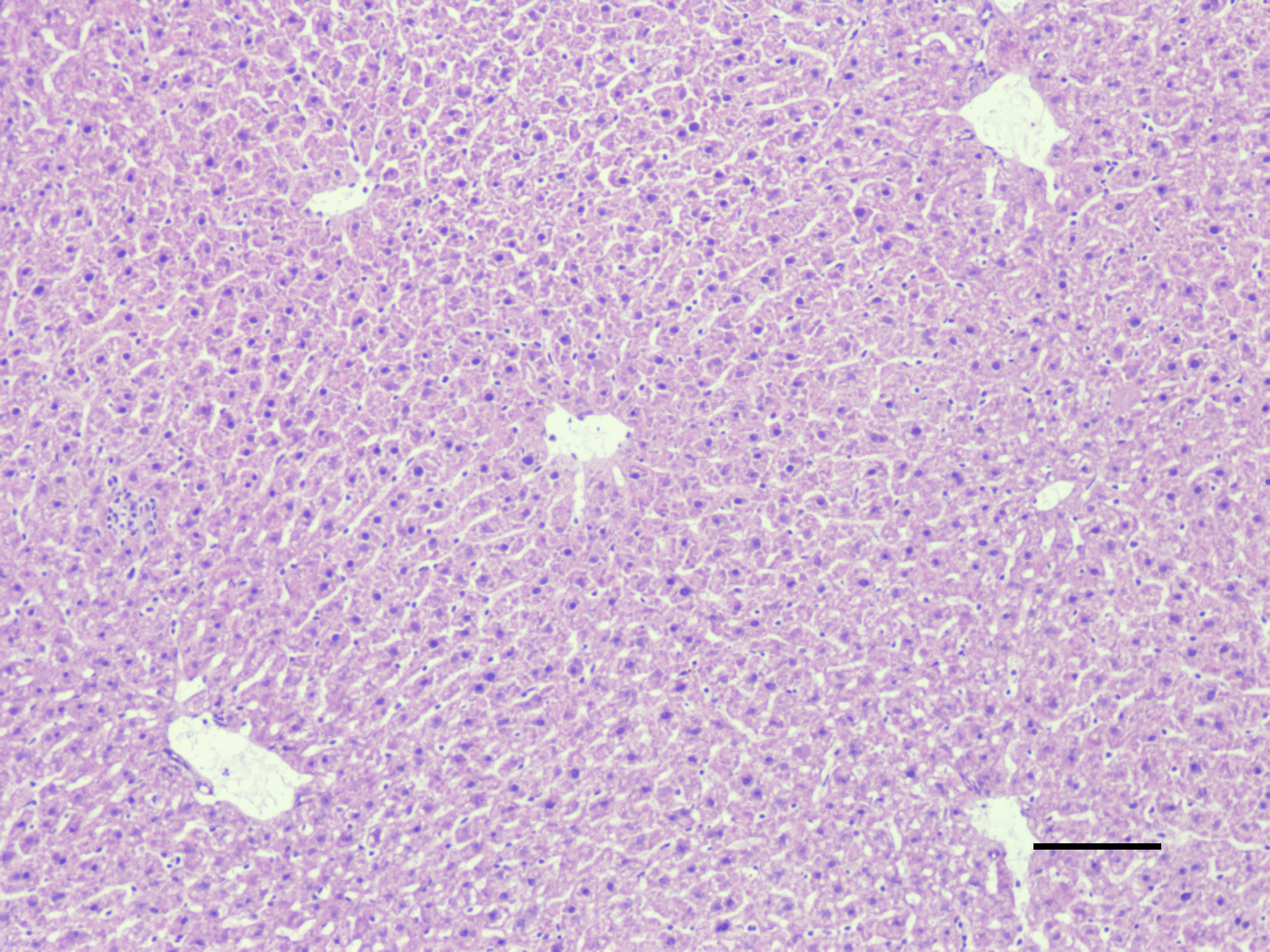

Supplement: Figure 4—figure supplement 1—source data 1. [file elife-92075-fig4-figsupp1-data1.zip › miR36_10x_ID_47_scale.jpg]
