# Supplementary material for: Liver microRNA transcriptome reveals miR-182 as link between type 2 diabetes and fatty liver disease in obesity
Source: eLife. 2024 Jul 22;12:RP92075. doi: 10.7554/eLife.92075 (PMC11262792; doi:10.7554/eLife.92075)
Supplement: Figure 2—source data 2. [file elife-92075-fig2-data2.pdf]

Figure 2 E (1/4): Chemiluminescence image of biological replicate 1 and 2

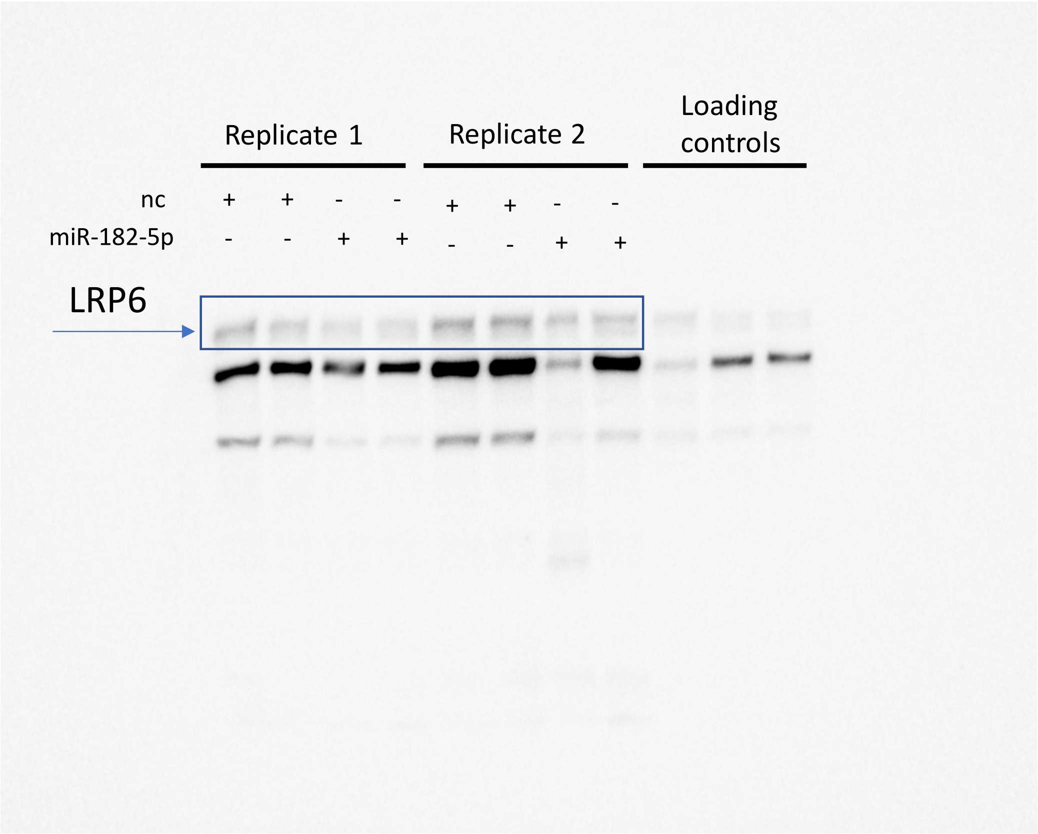

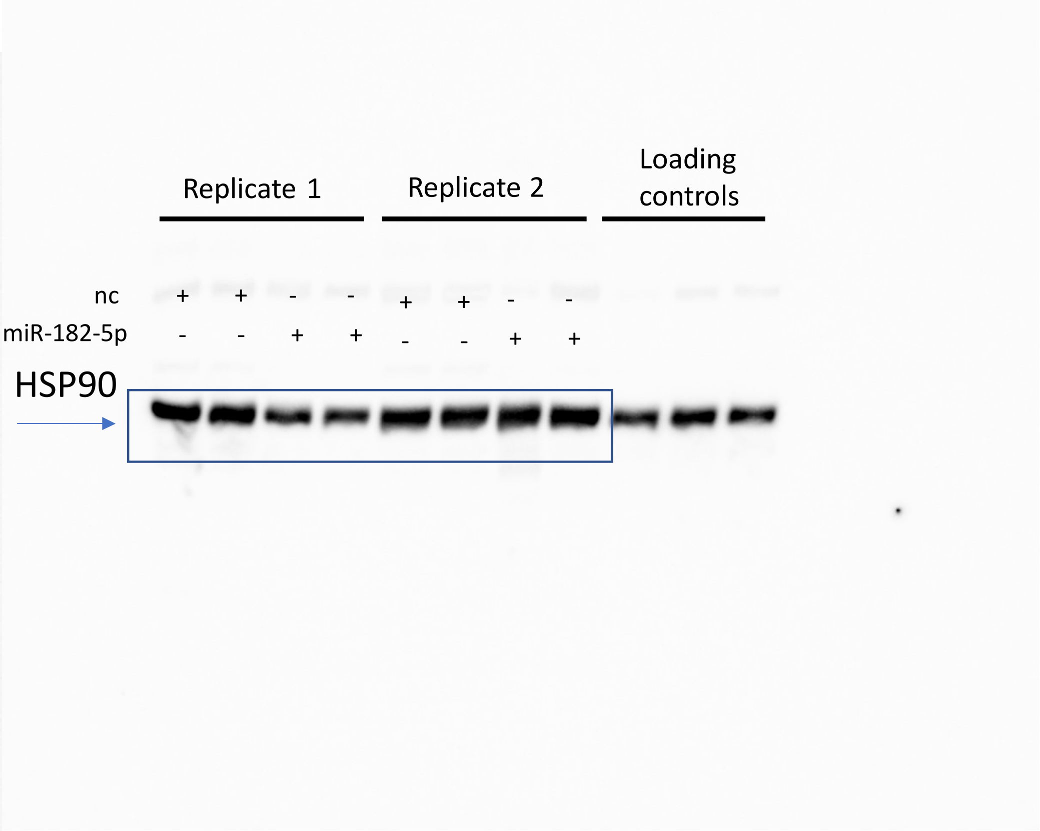

Parts of the blot used for quantification are indicated by a blue box

Figure 2 E (2/4): Multichannel image of replicate 1 and 2 with marker

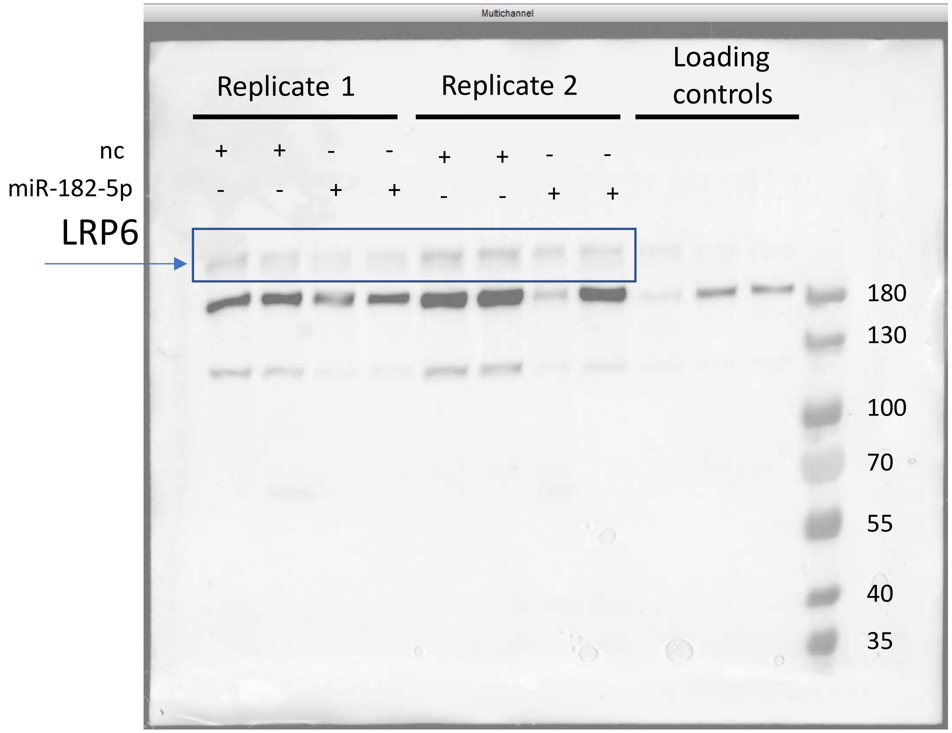

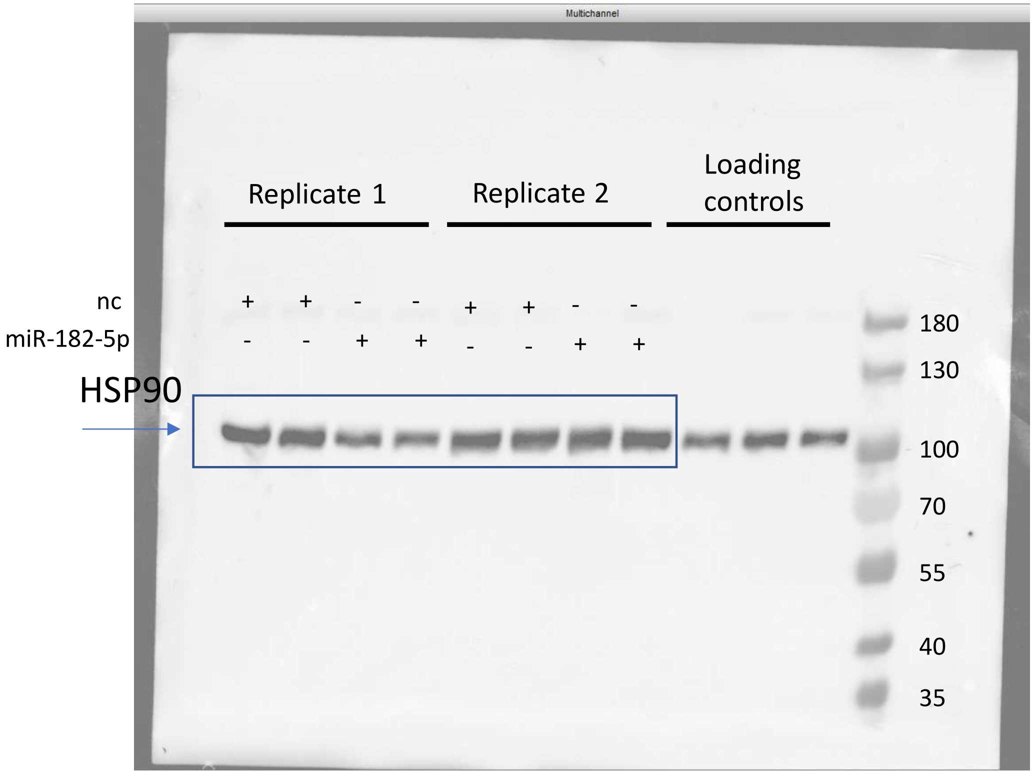

Parts of the blot used for quantification are indicated by a blue box

Figure 2 E (3/4): Chemiluminescence image of biological replicate 3

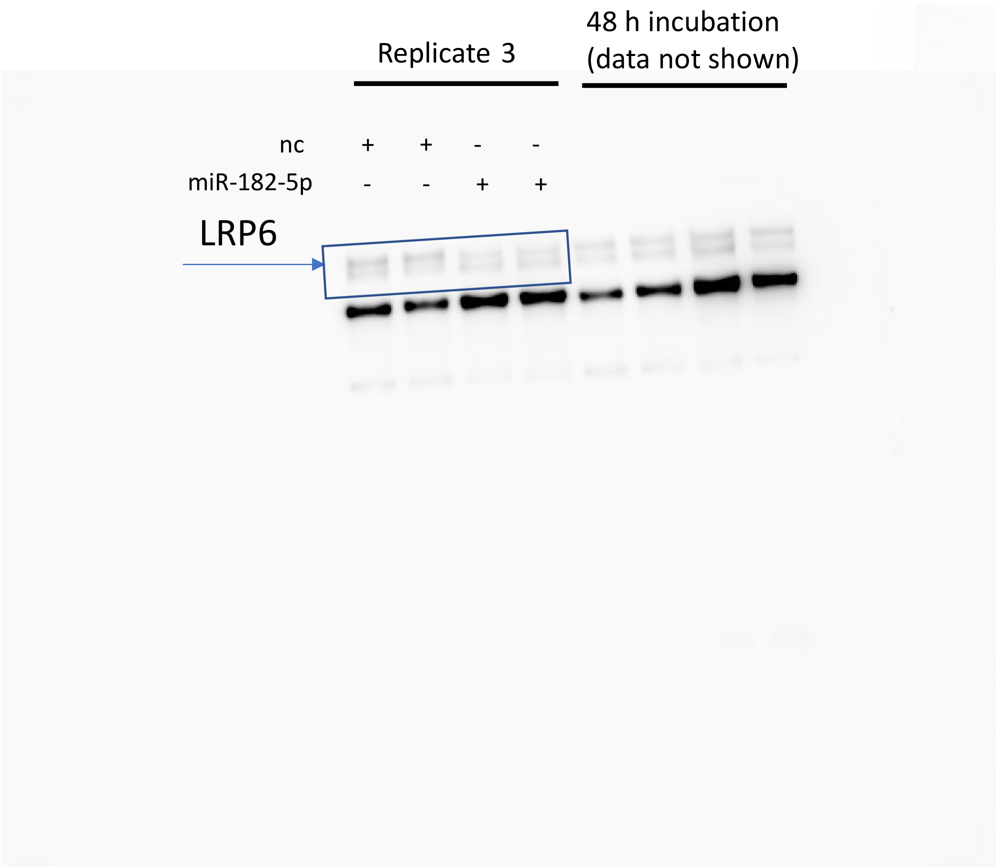

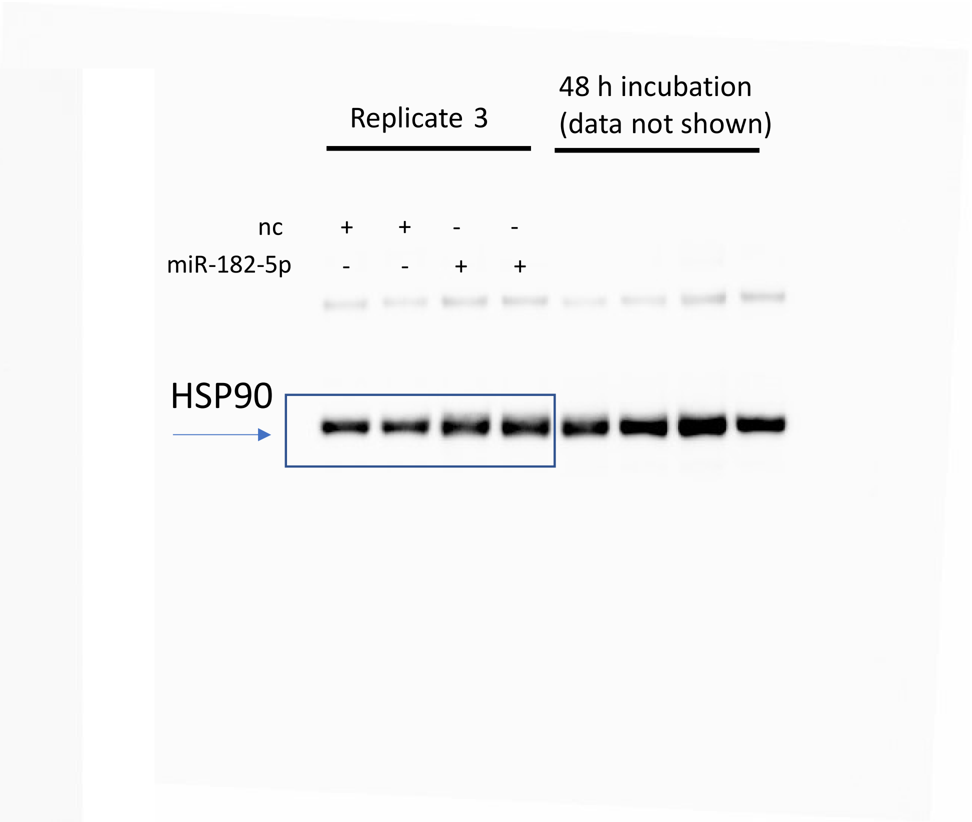

Parts of the blot used for quantification are indicated by a blue box

Figure 2 E (4/4): Multichannel image of replicate 3 with marker

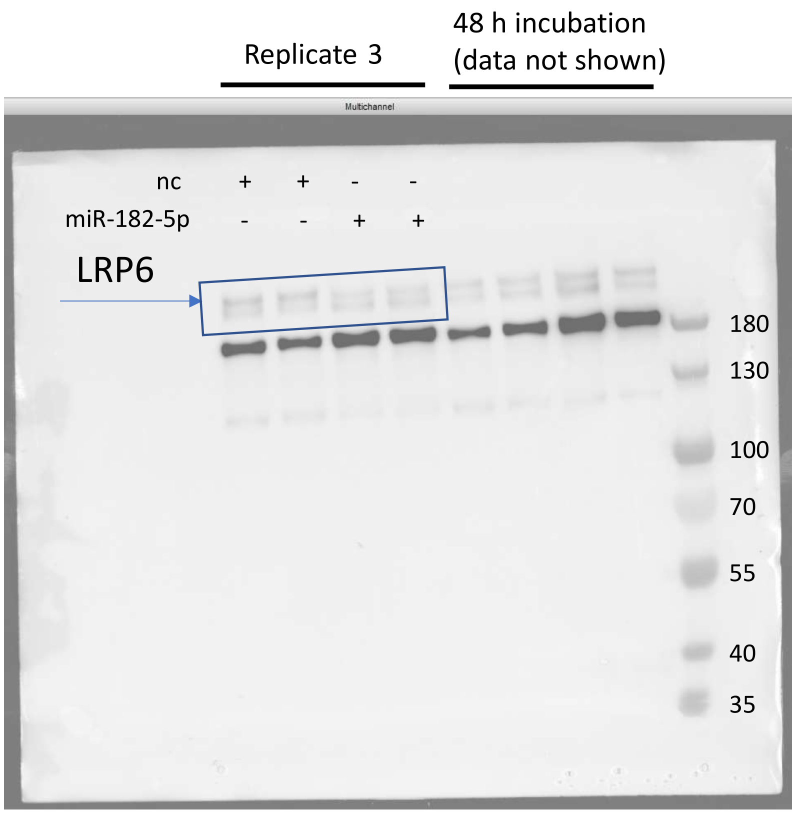

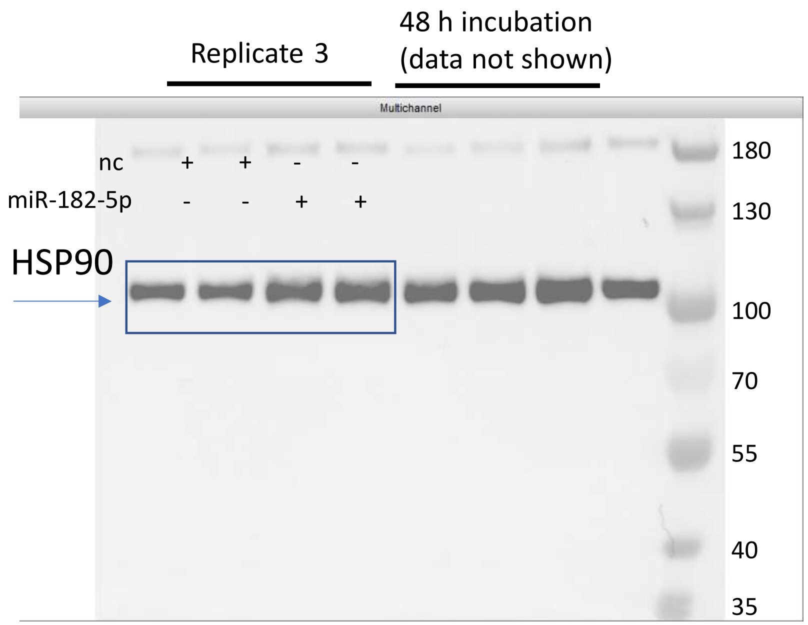

Parts of the blot used for quantification are indicated by a blue box
