# Supplementary material for: Liver microRNA transcriptome reveals miR-182 as link between type 2 diabetes and fatty liver disease in obesity
Source: eLife. 2024 Jul 22;12:RP92075. doi: 10.7554/eLife.92075 (PMC11262792; doi:10.7554/eLife.92075)
Supplement: Figure 2—source data 4. [file elife-92075-fig2-data4.pdf]

Figure 2 H (1/2):

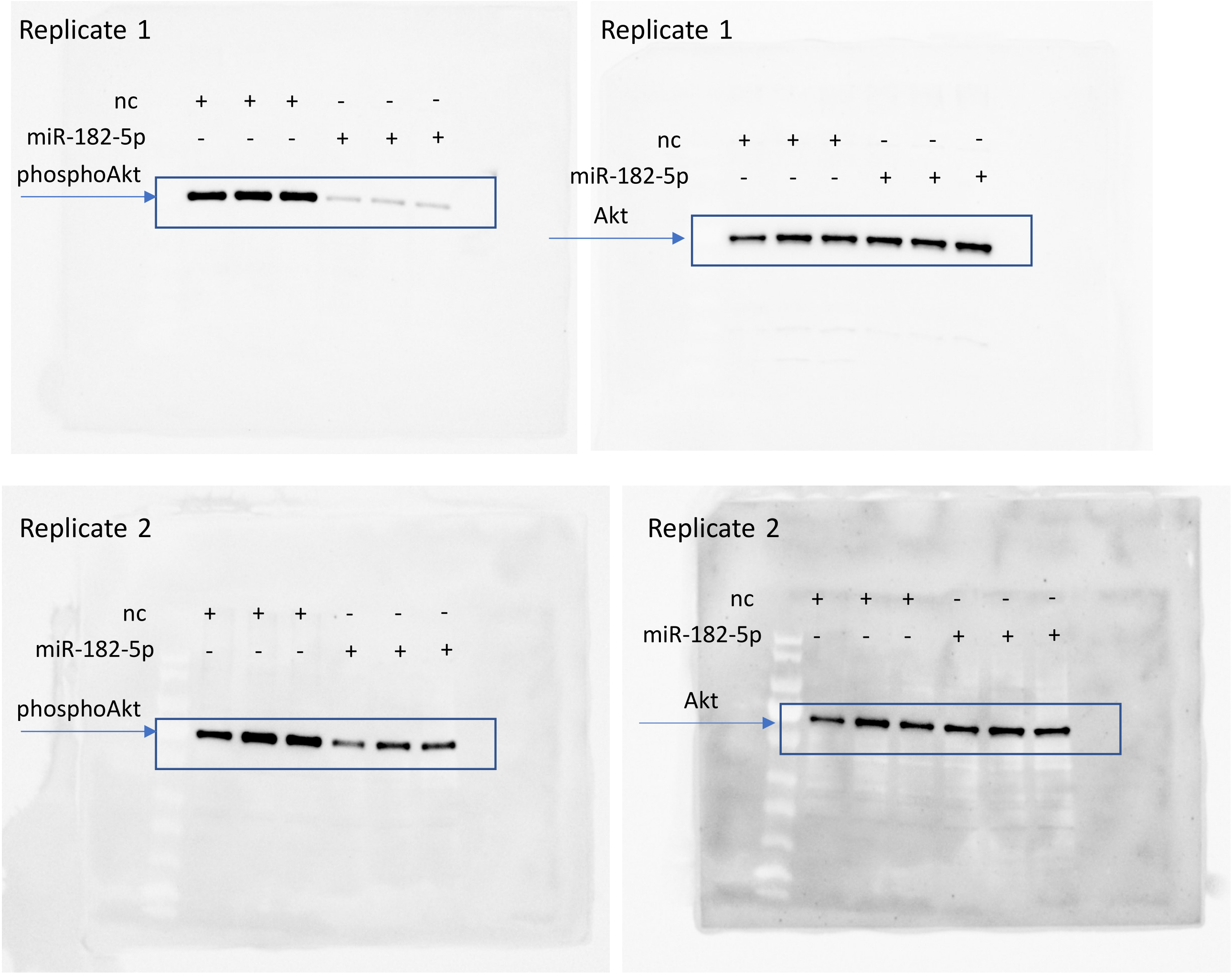

Parts of the blot used for quantification are indicated by a blue box

Figure 2 H (2/2):

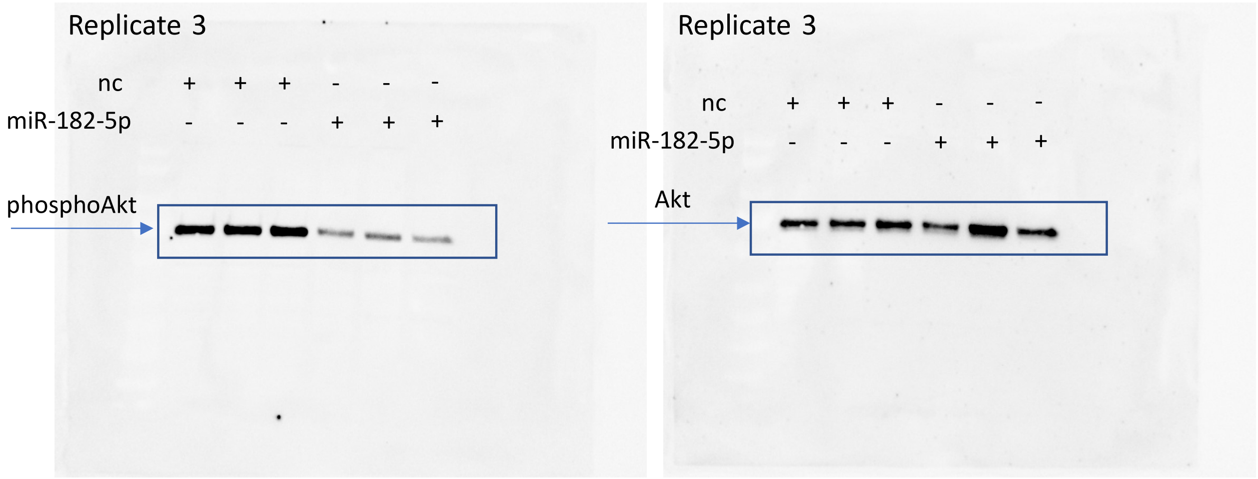

Parts of the blot used for quantification are indicated by a blue box
