# Supplementary material for: Liver microRNA transcriptome reveals miR-182 as link between type 2 diabetes and fatty liver disease in obesity
Source: eLife. 2024 Jul 22;12:RP92075. doi: 10.7554/eLife.92075 (PMC11262792; doi:10.7554/eLife.92075)
Supplement: MDAR checklist [file elife-92075-mdarchecklist1.docx]

Materials Design Analysis Reporting (MDAR)

Checklist for Authors

**Materials:**

| **Newly created materials** | **Indicate where provided:**  **section/figure legend** |
| --- | --- |
| N/A | N/A |

| **Antibodies** | **Indicate where provided:**  **section/figure legend** |
| --- | --- |
| Rabbit monoclonal anti-LRP6, Abcam, Cat#ab134146; RRID: AB_2895164 | Methods, 2.10 |
| Rabbit monoclonal anti-HSP90, Cell Signalling Technology, Cat#4877; RRID: AB_2233307 | Methods, 2.10 |
| Goat anti-rabbit HRP, Dako/Agilent, Cat#P0448; RRID: AB_2617138 | Methods, 2.10 |
| Rabbit monoclonal anti-Phospho-Akt (Ser473), Cell Signalling Technology, Cat# 4060; RRID: AB_2315049 | Methods, 2.10 |
| Rabbit polyclonal anti-Akt, Cell Signalling Technology, Cat# 9272; RRID: AB_329827 | Methods, 2.10 |

| **DNA and RNA sequences** | **Indicate where provided:**  **section/figure legend** |
| --- | --- |
| SYBR Green qPCR primer sequences | Supplementary Table 2 |
| TaqMan probes for qPCR (manufacturer IDs) | Supplementary Table 3 |
| Oligonucleotides for site-directed mutagenesis | Supplementary Table 2 |
| Primer for PCR amplification of LRP6-miR-182-5p recognition motif and subsequent cloning | Supplementary Table 2 |

| **Cell materials** | **Indicate where provided:**  **section/figure legend** |
| --- | --- |
| HepG2, ATCC, Manassas, US | Methods, 2.9 |
| HEK-293, ATCC, Manassas, US | Supplemental methods |

| **Experimental animals** | **Indicate where provided:**  **section/figure legend** |
| --- | --- |
| Male C57BL/6J mice, purchased at 5-6 week of age from Janvier Labs, Saint-Berthevin, Cedex, France | Methods, 2.12 |

| **Plants and microbes** | **Indicate where provided:**  **section/figure legend** |
| --- | --- |
| Escherichia coli, NEB® 5-alpha Competent E. coli (High Efficiency), New England Biolabs, Cat#C2987H | Supplemental methods |

| **Human research participants** | **Indicate where provided:**  **section/figure legend** |
| --- | --- |
| Men and women of West European ethnicity | Methods, 2.1 and supplemental table 1 and 2 for details |

**Design:**

| **Study protocol** | **Indicate where provided:**  **section/figure legend** |
| --- | --- |
| N/A | N/A |

| **Laboratory protocol** | **Indicate where provided:**  **section/figure legend** |
| --- | --- |
| N/A | N/A |

| **Experimental study design (statistics details)** | |
| --- | --- |
| **For in vivo studies: State whether and how the following**  **have been done** | **Indicate where provided:**  **section/figure legend. If it could**  **have been done, but was not,**  **write “not done”** |
| **Sample size determination** | Yes, methods 2.2 |
| **Randomisation** | Yes but not described |
| **Blinding** | Yes but not described |
| **Inclusion/exclusion criteria** | Yes but not described |

| **Sample definition and in-laboratory replication** | **Indicate where provided:**  **section/figure legend** |
| --- | --- |
| Cell culture experiments were replicated at least 3 times (biological replicates) from at leat two technical replicates. Sample sizes of biological replicates are shown in methods, tables and figure legends | Table and figure legends, methods |
| Human und murine liver data are shown as biological replicates | Table and figure legends, methods |

| **Ethics** | **Indicate where provided:**  **section/figure legend** |
| --- | --- |
| Human study approved by local ethics committee, PV4889. | Methods, 2.1 |
| Mouse study approved by the State of Bavaria, Germany. Experiments were performed at the Research Unit NeuroBiology of Diabetes, Institute for Diabetes and Obesity, Helmholtz Centre Munich. | Methods, 2.2 |

| **Dual Use Research of Concern (DURC)** | **Indicate where provided:**  **section/figure legend** |
| --- | --- |
| N/A | N/A |

**Analysis:**

| **Attrition** | **Indicate where provided:**  **section/figure legend** |
| --- | --- |
| All ΔCt-values which are not within a three standard deviations interval of all samples for the respective gene were defined as outliers and excluded for further analysis | General statistics of Supplemental methods |

| **Statistics** | **Indicate where provided:**  **section/figure legend** |
| --- | --- |
| Microarray statistics: Age, sex, BMI and NAS were considered as potential confounding factors for the altered expression of microRNAs and the incidence of T2D and therefore included as cofactors for logistic regression models. | Microarray statistics of methods, 2.13 and Supplemental methods |
| General Statistics: The data was normal distributed (tested by Lilliefors test) and therefore either a student’s t-test or One-way ANOVA was used to test significance. | General statistics of methods, 2.14 and Supplemental methods |

| **Data availability** | **Indicate where provided:**  **section/figure legend** |
| --- | --- |
| Microarray data is publicly available at GEO (human: GSE176025, mouse: GSE211367) | Methods, 2.16 |

| **Code availability** | **Indicate where provided:**  **section/figure legend** |
| --- | --- |
| Script code written in MATLAB and R (miRNA Nvis) is publicly available at https://github.com/christinkrause55/microRNA_network_visualizer | Methods, 2.16 |

**Reporting:**

| **Adherence to community standards** | **Indicate where provided:**  **section/figure legend** |
| --- | --- |
| The sectional reporting guidelines were used according to STROBE. | Methods, 2.1 |
